# Public engagement in the development of the National Health Insurance: a study involving patients from a central hospital in South Africa

**DOI:** 10.1186/s12889-020-09270-8

**Published:** 2020-07-31

**Authors:** Lizeka Amanda Tandwa, Ames Dhai

**Affiliations:** School of Clinical Medicine, Faculty of Health Sciences, University of the Witwatersrand, Johannesburg, South Africa

**Keywords:** National Health Insurance, Patients, Public engagement, Involvement

## Abstract

**Background:**

The National Health Insurance (NHI) is a proposed health policy in South Africa that aims to change the structure of the current health system. Public involvement in policy making is important and it is a constitutional requirement in the legislation development process in South Africa. Patients are key stakeholders and should be engaged in NHI policy process. Before patients can be engaged, they need to be provided with sufficient information about the NHI. Therefore, the aim of this exploratory study was to examine the levels of patient awareness of the NHI, which is a requisite for meaningful engagement.

**Methods:**

This was a cross sectional study of 244 patients from the follow-up clinics at the Department of Internal Medicine, Charlotte Maxeke Johannesburg Academic Hospital in the Gauteng Province, South Africa. The patients were interviewed using a structured interview process and a questionnaire. Descriptive statistics and logistic regression analyses were conducted.

**Results:**

The majority (79.51%) of the participants were not aware of the proposed National Health Insurance (NHI) in South Africa even though the NHI policy process commenced in 2011. Of the participants who were aware of the NHI, 86% responded that they had not been provided with an opportunity to be involved in the policy making process of the NHI. The odds of awareness were higher for male (OR: 2.08, 95% CI: 1.11–3.9, *p* value: 0.02) than female participants; White (OR: 2.36, 95% CI: 1.06–5.26, *p* value: 0.04) and Indian (OR: 2.76, 95% CI: 0.10–7.60, *p* value: 0.05) participants when compared to Black participants; and retired (OR: 3.13, 95% CI: 1.35–7.25, *p* value: 0.008) than unemployed participants.

**Conclusion:**

The levels of awareness of the NHI were low among the participants from Department of Internal Medicine, CMJAH. Without the awareness and information about the NHI, patients are not equipped to be involved in the NHI policy process in a meaningful way. Public patients are the intended beneficiaries of universal health coverage, therefore they should be prioritized in the NHI community engagement process to ensure that the NHI is community and patient centred.

## Background

The South African National Health Insurance (NHI) is health legislation that is under development. This legislation aims to ensure health equity through universal health coverage, and to address the burden of diseases in South Africa [1]. The NHI will achieve these aims through transforming the structure and the financing model of the South African healthcare system, and it is set to be implemented in phases over 14 years [1–3].

Public involvement in the law making process is a constitutional requirement in South Africa. Sections 59 (1) (a), 72 (1) (a) and 118 (1) (a) of the Constitution stipulate that the National Assembly and the National Council of Provinces (NCOP), the two houses of the Parliament of South Africa, together with the provincial legislatures must involve the public in legislative processes [4]. Public involvement is in consonant with the representative and participatory elements that the South African democracy is founded on [4]. The World Health Organisation (WHO) has identified key stakeholders of the health policy making process which include government and private sectors, community groups and importantly, patients [5]. These stakeholders ought to be engaged at the different stages of the policy process [4, 5].

Patient engagement is “to promote and support active patient and public involvement in health and healthcare and to strengthen their influence on healthcare decisions, at both the individual and collective levels” (pp.223) [6]. Patient engagement occurs at three levels: micro-engagement which is involvement and decision-making at the individual and clinical level; meso-engagement is decision-making at the organizational level and macro-engagement is patient involvement in policy decision-making at the district, national and international levels [6–8]. Patient engagement in the NHI is a type of macro-engagement. Patient engagement at all levels is important because decisions made at these levels have implications for the health and well-being of patients. Patients can be engaged individually or directly and indirectly, through designated representatives.

Patient engagement enhances patient dignity and autonomy, which is important for patient activation, i.e., the patient’s ability to have an active role in health. This has a positive influence on the health outcomes and experiences of patients in the health system [9]. The Ottawa Charter for Health Promotion recognizes that engagement empowers communities and patients to have a sense of ownership over their health lives [10].

Raboshakga proposed a two-step reasonableness approach to fulfil the constitutional requirement of public involvement [11]:

1. The public needs to be equipped with the relevant information on the policy or legislation and must be aware of their right to be involved in the policy making process. This promotes public awareness in the legislative process, prior to involvement.
2. An opportunity needs to be provided for interested members of the public to be involved in a meaningful and effective way [11].

The essential elements of this approach include receiving information, awareness of the right to be involved, galvanizing public interest and providing an opportunity for the public to be involved [11].

It was reported in the NHI White Paper that 150 written submissions were made and more than 60,000 citizens were engaged through national and provincial roadshows during the development of the NHI Green and White Papers [12]. Citizens were invited to make written and oral submissions, directly or through representatives, on each policy document when it was released [13]. Sixty thousand citizens at a national engagement level is only a small percentage of the South African adult population of 40.7 million people [14] and it is unclear who was engaged during these campaigns and whether the engagement was effective [12]. In August 2019, the NHI Bill was tabled in the Parliament of South Africa and stakeholders were invited to submit comments and participate in public hearings [15]. The NHI Bill was preceded by the NHI Green Paper in 2011, NHI White Paper in 2015 and the NHI Policy in 2017 [1, 12, 13, 16].

A few studies have investigated the broader community’s and healthcare practitioners’ awareness and perceptions of the NHI [17–20]. The aim of this exploratory study was to examine the levels of awareness among patients, as key stakeholders, from follow-up clinics at the Department of Internal Medicine, Charlotte Maxeke Johannesburg Academic Hospital (CMJAH). There was a specific focus on chronic patients as a sub-group of the broader community, because they are active public healthcare users and they are the intended beneficiaries of the NHI. This study also investigated whether the patients, who were aware of the NHI, had been involved in the NHI policy making process.

## Methods

This study was cross sectional and quantitative, utilizing structured interviews and was comprised of descriptive and comparative analyses.

### Study setting and sampling procedure

The study participants were patients from rheumatology, pulmonology and nephrology follow-up clinics at the Department of Internal Medicine, CMJAH. Charlotte Maxeke Johannesburg Academic Hospital is a central teaching hospital in the Gauteng Province and receives referrals from regional and tertiary hospitals in and around Gauteng Province [21]. Data was collected from 244 participants. The sample size was calculated using the StatCalc EpiInfo software. An 80% proportion of awareness was used for the calculation, based on a previous public awareness study on NHI in South Africa [18]. A 5% margin of error, with a confidence interval of 95% and an estimated population size of 20,000 patients per annum who access the medical services at the Department of Internal Medicine of CMJAH, was used.

Convenient sampling was used to recruit patients while they attended the follow-up clinics. They were individually approached and recruited and later enrolled into the study after obtaining written informed consent. Participants of all races and both males and females were enrolled. All participants had to be 18 years and older in order to be involved in the study. Participants were excluded from the study if they were under the age of 18 years and could not communicate in English, isiZulu and/or isiXhosa as the researcher was fluent in these languages.

### Data collection and instrument

The questionnaire had close-ended questions and included demographic questions; and questions on awareness on the NHI and involvement in the NHI policy making process. The awareness questions, were drawn from a questionnaire that was developed by the National Department of Health of South Africa that was used in another similar study [18]. Prior to the collection of data, a pre-test of the questionnaire was conducted, where 10 participants were interviewed at the same study site, to determine feasibility of the study. As a result of the pre-test, the questionnaire was amended to ensure appropriateness of the demographic variables and to improve the flow of the interview process. Data was collected between the months of July and October 2017.

**Fig. 1:**
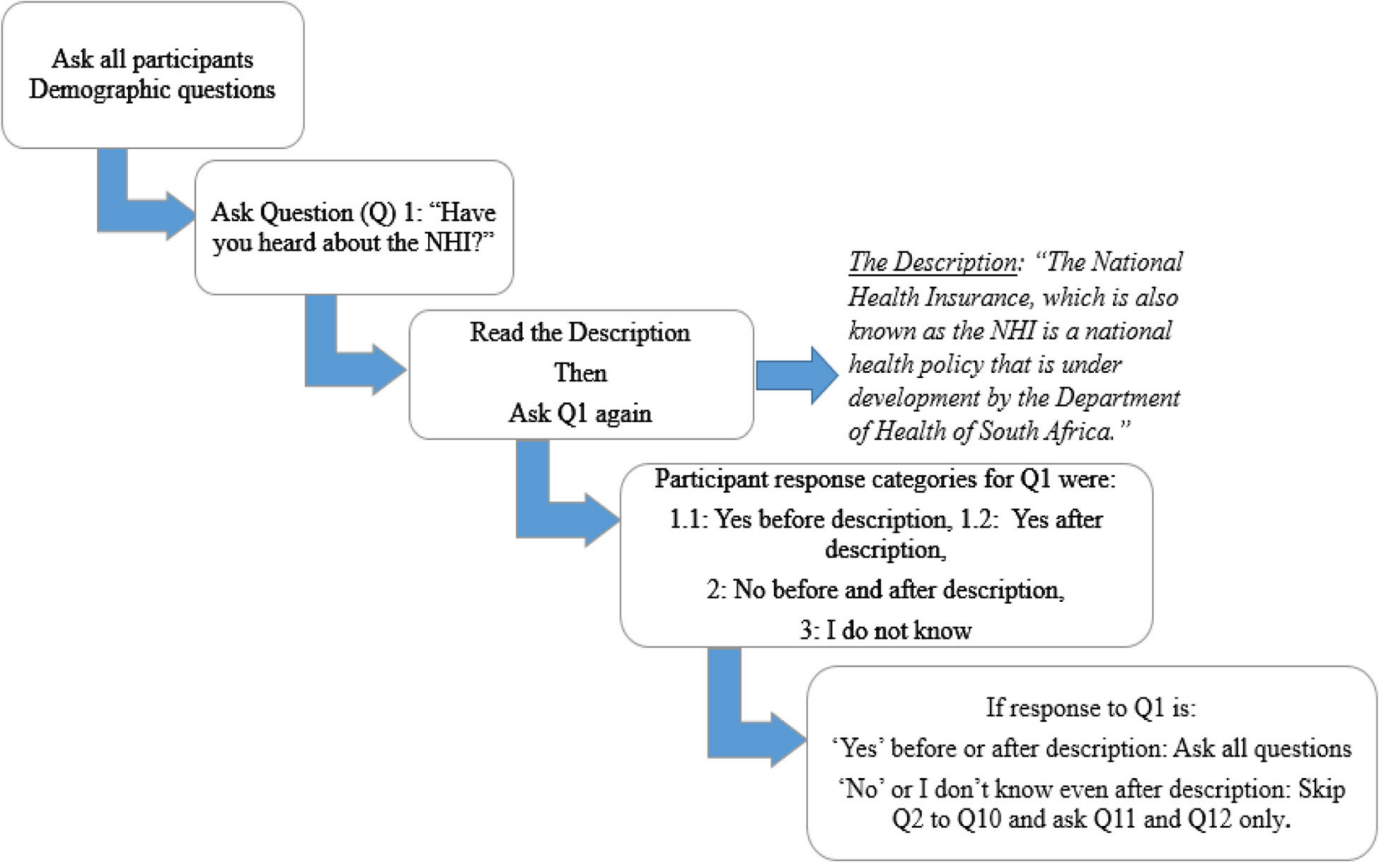
Figure depicting the structure and process of the interview

Demographic data collected included age, sex, race, employment status and education level. The questionnaire included questions with three answer options for each question. Participants who were aware of the NHI were asked all 12 questions and those who were not aware of the NHI were not asked questions 2–10 (Fig. 1). After the interview all participants received an information booklet on the NHI developed by the National Department of Health of South Africa as part of information sharing [22].

### Data management and analyses

In order to safeguard the confidentiality of information obtained from the participants the discrete and categorical data were coded to ensure anonymity and was coded using Microsoft Excel. Descriptive statistics for the demographic variables and the questionnaire answers were used for analysis. A Shapiro-Wilk test for normality was run for the age variable. Unadjusted and adjusted binary logistic regression tests were conducted to investigate if there were associations between the demographic (independent) variables and the levels of patient awareness of the NHI (dependent variable). STATA® software version 14 (College Station, TX: StataCorp LLC) was used to run the statistical analyses.

### Ethical considerations

Ethics approval to conduct this study was obtained from the Human Research Ethics Committee (Medical) (HREC) at the University of the Witwatersrand. The clearance number is: M1704105. Permission to conduct the study was obtained from the CMJAH Clinical Director and the Clinical Head of Department of the Internal Medicine.

## Results

### Demographics

The participant’s age range was between 18 and 82 years and the mean age was 41.49 years ±15.2 (mean ± SD). The age was normally distributed. There were more female participants in this study and most of the participants were Black (Table 1). Half of the participants were unemployed although of working age, and 13.93% of the participants were retired. Most of the participants had received secondary education and over one-third of the participants had received tertiary education.

**Table 1:** Participants’ Demographics (*n* = 244)

Variables	Frequency (n)	Percentage (%)
Sex		
Male	97	39.75
Female	147	60.25
Race		
Black	166	68.03
White	36	14.75
Coloured	18	7.38
Indian	19	7.79
Other	5	2.05
Employment status		
Employed	76	31.15
Semi-employed	12	4.92
Unemployed	122	50.00
Retired	34	13.93
Education level		
No education	1	0.41
Primary	13	5.33
Secondary	137	56.15
Tertiary	93	38.11

### Awareness on the NHI

A low proportion of the participants 20.49%, (*n* = 50) responded that they had heard about the NHI. Of those who were aware of the NHI, 19.67% replied that they had heard about the NHI before they were provided with a brief description of the NHI. Less than 1 % of the participants replied that they had heard of the NHI after the brief description was provided to them.

Table 2 depicts the results of the participants who replied yes to question 1: “Have you heard about the South African National Health Insurance (NHI)?”, with and without the description on the NHI. Of these participants (20.49%), 68% of them knew that the NHI would change the South African Healthcare sector. The fact that medical expenses would be covered by the NHI was known by 58, and 62% of the participants knew that the expenses would be paid for through the national budget. Sixty-four percent of the participants were aware that all citizens would have the same access to medical assistance through the NHI. However, 50% of these participants were not aware if both the employed and unemployed would receive the same access to medical services. A high proportion of these participants were aware that the NHI had been under discussion for many years and 60% had been provided with information about the NHI.

**Table 2:** Participants’ level of awareness of the NHI (*n* = 50)

Questions	Yes (%) (n)	No (%) (n)	I do not know (%) (n)
2. Will the NHI change the South African Healthcare sector?	68 (34)	18 (9)	14 (7)
3. Will the NHI pay for medical expenses?	58 (29)	18 (9)	24 (12)
4. Will the NHI be paid for from South African national budget?	62 (31)	14 (7)	24 (12)
5. Will everyone have the same access to medical assistance through the NHI?	64 (32)	28 (14)	8 (4)
6. Will both the employed and unemployed access healthcare through NHI?	50 (25)	36 (18)	14 (7)
7. Has the NHI policy been under discussion for many years?	76 (38)	4 (2)	20 (10)
8. Have you been provided with information about NHI?	60 (30)	40 (20)	
9. Can you participate/ be involved in the NHI policy discussions?	62 (31)	38 (19)	
10. Have you received an opportunity to participate/ be involved in the NHI policy discussions?	14 (7)	86 (43)	

### Involvement in the NHI policy development

Participants were asked if they had received an opportunity to be involved in the NHI policy making process. Of the participants that had heard about the NHI (20.49%, *n* = 50), 62% of them knew that they could participate in NHI policy discussion. Even though a high proportion (86%) of these participants had not received an opportunity to participate in policy discussion about the NHI.

All the participants (*n* = 244) were asked if they would be interested to participate in NHI policy discussions, and 84.43% of them said that they would be interested in being involved in the NHI policy making process. The majority of participants (91.39%) knew that they had a right to be involved in policy making process.

### Binary logistic regression

In order to run the logistic regression models changes had to be made to the data categories. The semi-employed and employed categories were combined into one category: ‘employed’. There was one participant who had no education and this data did not run in the logistic regression; therefore this category was removed. Therefore, employment status and education levels had three categories each for these analyses instead of the four as on the questionnaire.

The unadjusted logistic regression showed that the demographic variables that were significant predictors of awareness on the NHI were sex, race and employment status (Table 3). The odds of awareness of the NHI were 2.08 times higher among males compared to females. Compared to Black participants, the odds of awareness of the NHI were 2.36 times and 2.76 times higher in the White and Indian participants respectively. In addition, the odds of awareness of the NHI among the retired were 3.13 times higher compared to participants who were unemployed.

**Table 3:** Unadjusted logistic regression model for NHI awareness (*n* = 244)

Variables	Odds Ratio	95% Confidence Interval (CI)	*P*-value
Age (years)			
< 20 (base)	1	–	–
20–29	0.21	0.02–1.49	0.12
30–39	0.73	0.13–4.12	0.72
40–49	0.60	0.10–3.40	0.56
> 50	2.13	0.41–11.0	0.37
Sex			
Male	2.08	1.11–3.9	0.02*
Female (base)	1	–	–
Race			
Black (base)	1	–	–
White	2.36	1.06–5.26	0.04*
Coloured	0.28	0.04–2.17	0.22
Indians	2.76	0.10–7.60	0.05*
Other	1.18	0.13–10.96	0.884
Employment status			
Unemployed (base)	1	–	–
Employed	1.07	0.52–2.19	0.86
Retired	3.13	1.35–7.25	0.008*
Education level			
Primary (base)	1	–	–
Secondary	0.90	0.18–4.31	0.90
Tertiary	2.50	0.52–12.0	0.26

Base = 1.

*: *p* < 0.05 therefore statistically significant

When adjusting for sex, race, employment status and education level, the odds of awareness of the NHI were less among participants who were between the ages of 20–29 years when compared to participants who were younger than 20 years old (18–19 years) (Table 4). The odds of awareness for the sex, race and employment status variables decreased when adjusting for the confounding variables respectively, when compared to the unadjusted logistic regression model.

**Table 4:** Adjusted logistic regression model for NHI awareness (*n* = 244)

Variables	Odds Ratio	95% Confidence Interval (CI)	*P*-value
Age (years)			
< 20 (base)	1	–	–
20–29	0.12	0.02–1.03	0.053*
30–39	0.60	0.09–4.0	0.57
40–49	0.50	0.07–3.54	0.49
> 50	1.67	0.26–11.02	0.60
Sex			
Males	1.62	0.79–3.33	0.19
Females (base)	1	–	–
Race			
Black (base)	1	–	–
White	1.24	0.46–3.35	0.67
Coloured	0.19	0.02–1.61	0.13
Indians	1.40	0.42–4.63	0.60
Other	0.95	0.08–11.71	1.0
Employment status			
Unemployed (base)	1	–	–
Employed	0.88	0.38–2.04	0.77
Retired	1.14	0.37–3.46	0.82
Education level			
Primary (base)	1	–	–
Secondary	0.96	0.18–5.0	1.0
Tertiary	3.51	0.66–18.65	0.14

Base = 1.

*: *p* < 0.05 therefore statistically significant

## Discussion

The purpose of the present study was to explore the levels of patient awareness of the NHI and involvement in the policy making process. The data collection was conducted after the NHI Green Paper, NHI White Paper and NHI Policy were gazetted and open for public commentary, and preceded the tabling of the NHI Bill at the South African Parliament. The present study has four main findings. The fact that a high proportion of the participants had not heard about the South African National Health Insurance was the first and primary finding. The second finding is that the majority of the participants were aware that they have a right to be involved in the policy making process of the NHI. Thirdly, of those who were aware of the NHI (a minority), most of these participants responded that they had not received an opportunity to be involved in this policy making process. Finally, there were statistically significant associations between levels of patient awareness of the NHI and the sex, race and employment status demographic variables in the unadjusted logistic regression model.

The finding that the majority of the participants of the present study were not aware of the NHI was surprising, because the NHI Green Paper was released in 2011 and the present study was conducted 6 years later. A study conducted by Booysen, et al. also reported low levels of awareness of the NHI (13.3%) among public patients. However, Booysen, et al. collected their data in 2012, a year after the NHI Green Paper was gazetted [17]. Six years after the Green Paper, when the present study was conducted, it could have been assumed that the levels of awareness would had improved in specific sub-groups of the community, such as chronic patients.

The finding that the awareness levels of the NHI were low in the present study was inconsistent with other studies [18, 19]. Setswe, et al. found a much higher proportion (80.3%) of levels of awareness of the NHI, and their data was collected in 2013 [18]. This awareness percentage is virtually the inverse of what the present study found. The study by Setswe, et al. was conducted in three provinces in South Africa, with a combination of participants from rural, peri-urban and urban areas. Some of the participants in this study were from a NHI pilot site (Edendale Hospital in Umgungundlovu district). Even though awareness on the NHI was high in the study by Setswe et al., the majority of participants had limited understanding of important concepts of the national health insurance [18]. The difference in the awareness levels may be attributed to the difference in the sample population involved when compared to the present study. The present research focused on patients only and was conducted in a central hospital department, which is situated in an urban area, and it is not a NHI pilot site.

Raboshakga indicated that awareness of rights and stimulating public interest are both essential in ensuring that the public is involved in the policy making process [11]. In the present study, most of the participants knew that they have a right to be involved in the policy making process of the NHI. This positive finding is contrary to what was found in study conducted in a Tanzanian district, where the community members did not participate in the policy discussions because they were not aware that they had a right to be involved in policy decision-making [23].

Although the majority patients in the present study were aware of their right to be involved in the policy making process of the NHI, in order for the right to be involved in policy making to be realised, there ought to be fair opportunity [11] for patients to be involved in the policy making process, which the majority of the participants responded that they had not received. Pateman (2012) found that even though citizens may not be *au fait* with the technicalities of health policies, they are still interested in being involved [24]. This is because health policies affect their lives directly [24]. Pateman’s findings are consistent with the findings of this study because a majority of participants were interested in the policy making process. With higher levels of awareness of the NHI, interested stakeholders, can contribute to the policy development in a meaningful way.

The sex variable was a significant predictor for the levels of patient awareness of the NHI, and the odds of awareness were higher for male participants. A study on gender differences in the public perception of the South African NHI, reported that there were higher levels of support for the NHI among females [19]. Females are also considered to be more active users of the health system compared to males [25]. Contrary to the presumption for females to be more aware of the NHI, given previous studies’ findings [19, 25], the present study found that males higher odds of awareness of the NHI than females.

Race was also a significant predictor of awareness of the NHI, with White and Indian participants having higher odds of awareness on the NHI than Black participants, even though most users of the public health system and population in South Africa are Black citizens [12]. Much like the male participants, this indicates that White and Indian participants had access to information about the NHI, the sources of information that has influenced this result.

It is an interesting finding that unemployed participants who were of working age were 3 times less likely to be aware of the NHI than retired participants. The NHI would benefit and be of interest to unemployed and retired participants alike, since they are chronic patients. Therefore, unemployed participants would need to access the public health system as well because they do not have an alternative for health care.

Education plays an essential role in the levels of awareness of the NHI. The odds of awareness of those who had tertiary education being more than those who had primary education only in the unadjusted logistic regression model, although this finding was not significant. The odds of awareness of those with tertiary education compared to primary education were the highest in the multivariate analysis. Literature has shown that there is a directly proportional relationship between education level and awareness. The higher the education level, the more likely citizens are to have access to information and therefore have the ability to be involved in policy discussions [26–28]. A similar study on the awareness, knowledge and perceptions on the NHI found that the levels of support of the NHI were associated with the level of education, with higher education levels being associated with increased levels of awareness and support for the NHI [19].

### Limitations

The convenience sampling of the present study limits the generalizability of the study to all patients in South Africa. The follow-up clinics were chosen specifically because patients who continuously use the health facility’s follow-up clinics ought to be aware of the developments in the health system over time. Three major clinics at the Department of Internal Medicine were chosen instead of one, to allow spread of the participant pool, given the sampling methodology. This Department of Internal Medicine at CMJAH has an estimated 227 beds. This department alone is the same size as a medium-sized district hospital, which have 150 to 300 beds [21]. Language was a limitation because the researcher who conducted the interviews was only fluent in English, isiXhosa and isiZulu and the questionnaire was not officially translated into isiZulu and isiXhosa. The questionnaire was close-ended and did not have a follow-up question to establish what the involvement of those who had received an opportunity to be involved in the NHI policy process entailed.

## Conclusion

Most of the participants in this study were not aware of the NHI and therefore were not equipped with the necessary information to be involved in the policy making process in a meaningful way. Demographic groups who would be beneficiaries of the NHI given their reliance of the public health system, such as unemployed, Black (majority race group) and female participants had low levels of awareness. The drivers for access to information, thus awareness, among patients and the community need to be understood and targeted in order to involve key stakeholders in policy making and macro-engagement. A specific focus on previously disadvantaged groups and communities is important in the policy making process, to ensure a representative and patient-centred NHI.

## Supplementary information

**Additional file 1.** Study Questionnaire.

## Data Availability

Dataset used or analysed is available from corresponding author.

## Abbreviations

CMJAH: Charlotte Maxeke Johannesburg Academic Hospital
HREC: Human Research Ethics Committee (Medical)
NCOP: National Council of Provinces
NHI: National Health Insurance
Q: Question
WHO: World Health Organization
