# Supplementary material for: Public engagement in the development of the National Health Insurance: a study involving patients from a central hospital in South Africa
Source: BMC Public Health. 2020 Jul 31;20:1191. doi: 10.1186/s12889-020-09270-8 (PMC7393833; doi:10.1186/s12889-020-09270-8)
Supplement: Supplementary file 1 — Additional file 1. Study Questionnaire. [file 12889_2020_9270_MOESM1_ESM.docx]

**Study Questionnaire**

**Participant: ……….**

**Demographic information**

**Age**: **………**

**Sex**: Male□ Female□

**Race**: Black □ White□ Coloured□ Indian□ Other□

**Employment status**: Employed□ Semi-employed □ Unemployed□ Retired □

**Education Level**: No education □ Primary □ Secondary □ Tertiary □

**Questionnaire**

| **Questions** | **Yes** | | **No** | **I do not know** |
| --- | --- | --- | --- | --- |
| 1. Have you heard about the South African National Health Insurance (NHI)? | 1 | 2 |  |  |
| 2. Will the NHI change the South African Healthcare sector? |  | |  |  |
| 3. Will the NHI pay for medical expenses? |  | |  |  |
| 4. Will the NHI be paid for from South African national budget? |  | |  |  |
| 5. Will everyone have the same access to medical assistance through the NHI? |  | |  |  |
| 6. Will both the employed and unemployed access healthcare through NHI? |  | |  |  |
| 7. Has the NHI policy been under discussion for many years? |  | |  |  |
| 8. Have you been provided with information about NHI? |  | |  |  |
| 9. Can you participate/ be involved in the NHI policy discussions? |  | |  |  |
| 10. Have you received an opportunity to participate/ be involved in the NHI policy discussions? |  | |  |  |
| 11. Would you be interested to participate/ be involved in the NHI policy discussions? |  | |  |  |
| 12. Do you have a right to participate/be involved in the policy making process? |  | |  |  |

**1**: Participant replied “Yes” without a description. **2** Participant replied “Yes” after a description.
